# Eliminating viscosity bias in lateral flow tests

**DOI:** 10.1038/s41378-021-00296-5

**Published:** 2021-09-06

**Authors:** Daniel M. Kainz, Bastian J. Breiner, Susanna M. Früh, Tobias Hutzenlaub, Roland Zengerle, Nils Paust

**Affiliations:** 1 Laboratory for MEMS Applications, IMTEK - Department of Microsystems Engineering, University of Freiburg, Georges-Koehler-Allee 103, 79110 Freiburg, Germany; 2 Hahn-Schickard, Georges-Koehler-Allee 103, 79110 Freiburg, Germany

**Keywords:** Engineering, Physics

## Abstract

Despite the widespread application of point-of-care lateral flow tests, the viscosity dependence of these assay results remains a significant challenge. Here, we employ centrifugal microfluidic flow control through the nitrocellulose membrane of the strip to eliminate the viscosity bias. The key feature is the balancing of the sample flow into the cassette of the lateral flow test with the air flow out of the cassette. A viscosity-independent flow rate of 3.01 ± 0.18 µl/min (±6%) is demonstrated for samples with viscosities ranging from 1.1 mPas to 24 mPas, a factor greater than 20. In a model human IgG lateral flow assay, signal-intensity shifts caused by varying the sample viscosity from 1.1 mPas to 2.3 mPas could be reduced by more than 84%.

## Introduction

Lateral flow tests (LFTs) are one of the most common point-of-care tests. A variety of different samples can be used, such as whole blood^1^, serum^2^, urine^3^, saliva^4^, milk^5^, and many more^6–8^. The strips consist of different porous materials that are glued in series on a backing card. The applied sample is wicked through the strip by capillary forces. Each lateral flow strip has a functionalized site on the chromatographic membrane, generally in the form of a test line. At that site, binding reactions take place between analytes and detection molecules. The formation of the test line on the strip signifies the presence or absence of an analyte in the sample. LFTs often allow qualitative or semiquantitative readouts with the naked eye and are thus a versatile tool for field applications.

For some biomarkers, such as inflammation markers, quantitative determination is necessary for deciding on the appropriate medical treatment or for monitoring a therapy^9^. However, the development of quantitative LFTs is challenging, especially for samples with varying viscosities. Different sample viscosities lead to different wicking speeds and thus different flow rates through the strip. Inconsistent flow rates due to varying sample viscosities in turn lead to varying incubation times for the molecules at the binding sites. The consequence is viscosity bias in the signal intensities and thus over- or underestimations of the analyte concentrations. For that reason, varying sample viscosities is one of the challenges for LFTs^10^.

Biological samples can show large variations in viscosity. For example, the shear rate-dependent viscosity of saliva can range from 1.5 mPas to 23 mPas^11^. Although the viscosity of saliva can be equalized by a freezing step or by magnetic bead beating, the viscosities of the processed samples still vary between 1.5 mPas and 3 mPas^12^. Moreover, the viscosity of other common sample matrices, such as blood plasma, can also vary between 1.50 mPas and 1.72 mPas at 25 °C^13^. In addition, various diseases can alter plasma viscosity^14^. Even urine shows viscosity variations between 0.635 mPas and 0.797 mPas at 37 °C, depending on the fraction of solutes^15,16^.

Generally, varying sample viscosities can be equalized by using a dilution buffer with a specific viscosity. However, the additional liquid-handling step of prediluting the sample reduces the sensitivity and is therefore not feasible for every LFT application. Hence, flow control is essential for the development of highly quantitative and sensitive LFTs^17,18^. Nevertheless, capillary-driven flow controls are usually limited to only reducing the flow rate through the strip, for example, by increasing the fluidic resistance^19^ or by surface modification^20^.

A viscosity-independent flow without sample dilution can be achieved in capillary tubes by introducing a fluidic resistance for the outflowing air that overcomes the viscous dissipation of sample flow in the capillary system^21,22^. Since this approach depends highly on the surface energy of the sample, Guo et al. introduced a modified capillary pumping system that was independent of the liquid sample’s viscosity and surface energy^23^. A pump liquid was enclosed at the end of a glass capillary and was wicked out of the glass capillary through a flow restrictor and onto an absorbent pad. The sample liquid was drawn from the other side into the glass capillary, and because the outflow of the pumping liquid was defined, the resulting sample inflow was independent of the sample viscosity. Although the capillary-system approach is promising, transferring it to established LFTs is, in our opinion, challenging and to the best of our knowledge, it has not been shown before. The main challenge is likely achieving a tight seal between the LFT membrane and the capillary to avoid the sample flowing around the enclosed LFT, which would result in increased viscous liquid dissipation in the tube. In addition, the flow rate can only be adjusted by changing the capillary system or the pump liquid.

Another possibility is the use of centrifugal flow control^24–27^, for which leak-tight integration of the strip is not required. A further advantage of using centrifugal microfluidics is that various sample-preparation steps, such as blood plasma separation or sample dilution, can be integrated into a centrifugal cassette to enhance the usability^28–31^. The LFT is performed under rotation. Since the centrifugal force can be scaled by rotational speed, the flow through the strip can be adjusted precisely without changing the porous components. However, in all previously presented centrifugal solutions, the liquid-flow rate depended on the sample viscosity. Hence, the flow rate cannot be fine-tuned without measuring the sample viscosity.

Here, we present a centrifugal microfluidic cassette to facilitate viscosity-independent liquid flow through a conventional LFT nitrocellulose membrane. The key feature is centrifugal-driven pneumatic flow control, which was previously only shown with a comparable pneumatic concept in capillary tubes^21,22^. By balancing the sample flow into the cassette with the airflow out of the cassette, the flow rate becomes independent of the sample viscosity. The flow rate is controlled only by centrifugal forces and not by capillary forces, which are the traditional means of controlling the flow rate. This method enables easy external scaling of flow rates over a wide range, making the centrifugal cassette a versatile tool for established LFTs. In the following report, we explain in detail the working principle of the centrifugal cassette. We then experimentally analyze the flow rates of liquids with different viscosities through our cassette and compare centrifugation driven with capillary-driven pneumatic flow control. By using a model LFT assay, we show the viscosity bias and demonstrate for the first time experimentally how it can be eliminated by employing pneumatic flow control in the centrifugal cassette.

## Results

### Concept and fabrication of the centrifugal cassette

**Fig. 1:**
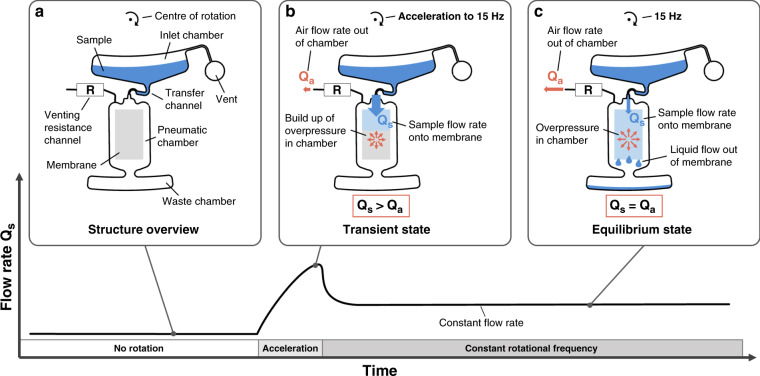
Schematic illustration of the working principle of the centrifugal cassette from no flow to a constant flow rate Q_s_. **a** Schematic illustration of the microfluidic design inside the cassette. **b** The rotational frequency is accelerated to 15 Hz. The sample starts flowing into the pneumatic chamber with the membrane inside. This sample inflow causes an overpressure that acts against the sample inflow. With increasing overpressure in the chamber, air starts flowing out of the pneumatic chamber through the venting resistance channel. **c** At equilibrium, the flow rates of both the sample inflow and the air outflow through the resistance channel are equal and the overpressure in the chamber remains unchanged. This is the actual operating state of the structure

A schematic overview of the microfluidic structure in the centrifugal cassette is depicted in Fig. 1a. Sample-preparation steps were omitted in the centrifugal cassette to maximize comparability with the dipstick assay. The centrifugal cassette for the LFT consists of two chambers: a vented inlet chamber and a pneumatic chamber with the membrane inside. The two chambers are connected by a transfer channel. The built-in siphon in the transfer channel prevents air backflow into the inlet chamber. The pneumatic chamber is vented only via a resistance channel. The transfer channel has an extremely low fluidic resistance compared with the venting resistance channel.

Under rotation, the sample flows from the inlet chamber through the transfer channel and into the pneumatic chamber. Due to the negligible fluidic resistance of the transfer channel, the sample flow rate is high at the beginning. Since the pneumatic chamber is only vented via a resistance channel, a pneumatic pressure builds up, as shown in Fig. 1b. The overpressure acts against the inflow coming from the transfer channel. This leads to a reduction in the flow rate in the transfer channel. At the same time, the outflow of air through the venting resistance channel increases with rising overpressure.

If rotation continues at a constant frequency, a state of equilibrium is reached, as shown in Fig. 1c. At equilibrium, the sample flow rate through the transfer channel is dictated by the maximum airflow rate through the venting resistance channel at a certain rotational velocity. The flow rate can thus be adjusted by dimensioning the venting resistance channel and by changing the rotational speed. Since the sample inflow is controlled by the outflow of air through the venting resistance channel, the sample inflow depends on the viscosity of air, and the sample viscosity has a negligible effect.

An important design consideration of the centrifugal cassette is the integration of the membrane. In a centrifugal system, the actuating forces and thus the flow rate can be adjusted precisely by regulating the rotational frequency. The sample must flow through the membrane completely to guarantee the best assay performance. When any part of the sample flows past the membrane rather than through it, it is called bypass. Bypass may occur if the fluidic structure is incorrectly designed. To prevent bypass flows, the inflow into the pneumatic chamber must be less than or equal to the maximum possible flow rate through the membrane at a certain frequency. At the maximum possible flow rate, the pressure drop due to liquid flow through the membrane is equal to the centrifugal pressure of the liquid column in the membrane. A further increase in flow rate would result in bypass because the centrifugal pressure would not be sufficiently high to transport all the liquid through the membrane. According to our recently published design guidelines for preventing bypass^26^, the maximum flow rate Q_M,max_ can be calculated as follows:

1 $$Q_{M,\max } = \frac{{\rho _s \ast r_{in,M} \ast \omega ^2 \ast A_M \ast \kappa }}{{\eta _s}}$$

where *ρ*_*s*_ denotes the density of the sample, *r*_*in,M*_ is the inner radial position of the membrane, *ω* is the angular frequency, *A*_*M*_ is the cross-sectional area of the membrane, *κ is* the permeability of the membrane, and *η*_*s*_ is the viscosity of the sample. According to Eq. (1), *Q*_*M*,max_ is 6.43 µl/min for the 1.1-mPas sample and 3.12 µl/min for the 2.3-mPas sample in the centrifugal cassette at 24 °C and at 15 Hz. If higher flow rates are needed, the rotational frequency must be increased, as shown in a previous study^26^. The centrifugal pressure Δ*p*_cent_ of the sample liquid column is the driving force in the system and can be calculated with the following equation:

2 $${\Delta}p_{{{\rm{cent}}}} = \frac{{\rho _s}}{2}\omega ^2\left( {r_2^2 - r_1^2} \right)$$

The centrifugal pressure was calculated as 13.7 hPa at 15 Hz for a sample with a density of 1000 kg/m³.

**Fig. 2:**
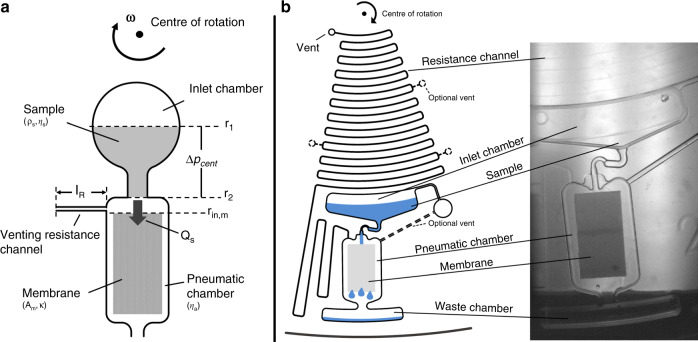
Overview of the centrifugal cassette with pneumatic flow control. **a** Schematic illustration of the structure with all relevant parameters. **b** Left: Structure overview; the optional vents were not used for the experiments in this study. Right: Stroboscopic picture of the thermoformed cassette during the experiment at 15 Hz

By balancing the centrifugal pressure of the sample liquid column with the viscous dissipation of the air outflow through the venting resistance channel, the viscosity-independent sample flow rate *Q*_*s*_ through the transfer channel can be calculated as a function of frequency:

3 $$Q_s = \frac{{\frac{{\rho _s}}{2}\omega ^2\left( {r_2^2 - r_1^2} \right)}}{{C_g\frac{{\eta _al_R}}{{A_R^2}}}}$$

where *ρ*_*s*_ denotes the density of the sample, *ω* is the angular frequency, *r*_2_ is the outer radial position of the channel, *r*_*1*_ is the inner radial position of the liquid column in the inlet chamber, *C*_*g*_ is the geometry factor of the channel, *η*_*a*_ is the viscosity of air, *l*_*R*_ is the length of the venting resistance channel, and *A*_*R*_ is the cross-sectional area of the venting resistance channel. The measured densities of the buffers varied slightly: 1.021 g/ml (1.1 mPas), 1.037 g/ml (2.3 mPas), and 1.092 g/ml (24 mPas). The calculated *Q*_*s*_ at 15 Hz and the above densities were 3.02 µl/min for the 1.1 mPas sample, 3.07 µl/min for the 2.3-mPas sample, and 3.22 µl/min for the 24 mPas sample. The expected flow rates were below the maximum possible flow rates through the membrane *Q*_*M*,max_; these expected flow rates were 6.43 µl/min for the 1.1-mPas sample and 3.12 µl/min for the 2.3-mPas sample. Thus, bypass-free flow through the membrane in the centrifugal cassette was assured. The calculated *Q*_*M*,max_ values also show that the highly viscous sample restricts the maximum possible flow rate through the membrane. All relevant parameters for the calculations are depicted in Fig. 2a, and a detailed derivation of the formulas can be found in the ESI.

The fabricated centrifugal foil cassette had three identical pneumatic flow-control structures. Each structure consisted of a vented inlet chamber, a transfer channel, a pneumatic chamber with the membrane, and a venting resistance channel, as shown in Fig. 2b. In addition to the venting resistance channel, the pneumatic chamber had an optional venting channel, which was not used in the experiments.

### Viscosity-independent flow-rate analysis

The key feature of the centrifugal cassette is that it balances the sample flow into the pneumatic chamber with the airflow out of that chamber. Hence, the sample flow rate depends on the viscosity of air, and the sample viscosity has a negligible effect. A PBS-based sample buffer was used to test the functionality of this viscosity-independent flow in the centrifugal cassette. To simulate different viscosities in biological samples, the sample buffer was mixed with PEG1000 to achieve viscosities of 1.1 mPas, 2.3 mPas, and 24 mPas. The centrifugal cassettes were processed at a constant rotational frequency of 15 Hz.

**Fig. 3:**
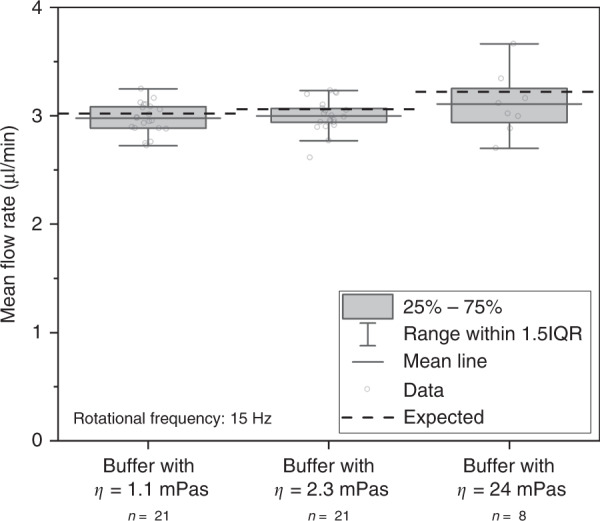
Measured flow rates of samples with viscosities of 1.1 mPas, 2.3 mPas, and 24 mPas at 15 Hz. The mean value is indicated with a line, and the density-dependent expected flow rate for each sample is shown with a dashed line. Each gray circle represents a measured value

The measured flow rates were independent of the sample viscosity. During the acceleration phase, 3.84 ± 0.24 µl (mean ± SD) of sample was transferred into the pneumatic chamber, creating the initial overpressure. The mean flow rate of all samples was 3.01 ± 0.18 µl/min at a rotational frequency of 15 Hz. The sample buffer with a viscosity of 1.1 mPas showed a mean flow rate of 2.98 ± 0.14 µl/min (mean ± SD) (CV = 5.0%). Increasing the viscosity to 2.3 mPas resulted in a mean flow rate of 3.00 ± 0.15 µl/min (CV = 5.0%), and increasing the viscosity by a factor of 22–24 mPas resulted in a mean flow rate of 3.11 ± 0.29 µl/min (CV = 9.5%). The statistical evaluation showed no significant differences (*p* = 0.198) in the mean flow rates between the three samples with different viscosities. All the flow rates of the 1.1-mPas and 2.3-mPas samples measured during the immunoassay experiment, as well as those of the 24-mPas samples, are plotted in Fig. 3.

### Viscosity bias of LFTs and its elimination

**Fig. 4:**
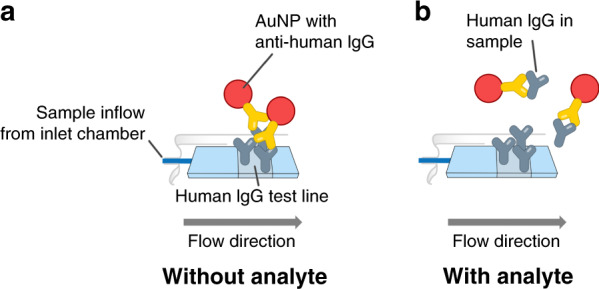
Schematic concept of the model human IgG lateral flow assay. **a** Without analyte (human IgG) in the sample, the conjugated AuNPs only bind to the human IgG on the test line. **b** With the analyte in the sample, the conjugated AuNPs also binds to human IgG in solution, and less conjugated AuNPs binds to the test line. Hence, the test line shows a weaker signal

Quantitative results from standard LFTs are biased due to the varying sample viscosities because the resulting variation in flow rates leads to different incubation times for molecules at the LFT-binding sites. This phenomenon leads to variation in the test-line signal development and thus to inaccurate quantitative results. To demonstrate the viscosity bias of an LFT, a human IgG model assay was used. The human IgG assay is a competitive immunoassay. Its working principle is depicted in Fig. 4. The membrane was functionalized with a human IgG test line. The sample was mixed with gold nanoparticles (AuNPs) as detection particles. The AuNPs had anti-human IgG on the surface. As the sample passed the test line, the antihuman IgG bound to the human IgG, and the test line formed, as shown Fig. 4a. If human IgG is present in the sample (analyte), it competes with human IgG on the test line for the binding sites of the antihuman IgG antibody, as shown in Fig. 4b.

The assay was performed as a lateral flow dipstick assay using sample buffers with 1.1 mPas and 2.3 mPas. To eliminate the viscosity bias of the LFT, the assay was simultaneously performed in the centrifugal cassette with pneumatic flow control. The test-line signal intensities of the dipstick LFT and of the assay in the centrifugal cassette were compared.

**Fig. 5:**
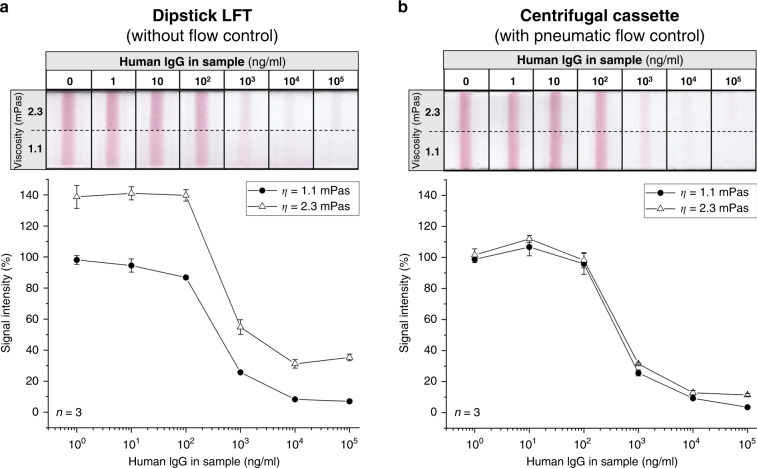
Scanned test lines of lateral flow strip assays that were performed as dipstick assays (a) and in the centrifugal cassette (b). The quantitative analyses of all test-line signal intensities are shown below. For better comparison, the signal intensity is defined as the relative intensity compared to the signal intensity of the negative control with a sample viscosity of 1.1 mPas. **a** The increase in the viscosity of the sample resulted in a mean signal increase of 38 percentage points. This difference can be seen with the naked eye, especially at high and low concentrations. **b** Using the centrifugal cassette, the mean signal increase was reduced to only six percentage points

The scanned test lines are shown in Fig. 5, and the quantitative results of the test-line peak analysis are plotted. All four curves show a similar pattern, with a high signal-intensity plateau up to 100 ng/ml and a low signal intensity plateau starting at approximately 10,000 ng/ml. In Fig. 5a, the results of the dipstick LFT are plotted. The signal intensity of the 2.3-mPas sample buffer is on average 38 percentage points higher than that of the sample buffer with a viscosity of 1.1 mPas.

In Fig. 5b, the results of the centrifugal cassette with pneumatic flow control are plotted. No bypass was observed in the centrifugal cassette. According to the flow-rate measurements illustrated in Fig. 3, there was no difference in the flow rates for different viscosities when the centrifugal cassette was used, and hence no difference in the incubation times of the 1.1 mPas and the 2.3-mPas sample buffers was observed. The signal-intensity shift between the high- and low-viscosity sample buffers could be reduced to only six percentage points on average, a more than 84% reduction compared with the results of the dipstick LFT.

## Discussion

### The elimination of viscosity bias in LFTs

In general, the signal development of the human IgG assay used shows a dependence on incubation time. With a higher viscosity, the capillary-driven flow rate through the dipstick was reduced. In our experiments, we measured a mean signal increase of 38 percentage points when we doubled the sample viscosity from 1.1 mPas to 2.3 mPas. This signal increase can be explained by an increase in the actual incubation time of the molecules at the binding sites on the test line. Longer incubation times result in higher signals and vice versa. Consequently, the requirements for a quantitative LFT in terms of sensitivity, accuracy, and precision are generally difficult to achieve if the sample viscosity is strongly patient dependent.

Compared with conventional LFTs, the centrifugal cassette with pneumatic flow control reduced the viscosity bias by 84 %. The remaining signal increase of six percentage points in the higher-viscosity sample cannot be explained by an increase in the incubation time because no significant difference in the flow rate was measured (see Fig. 3). We assume that PEG1000 used to increase the viscosity interferes with the binding process at the test line. In preexperiments (see ESI), we did not observe a nonspecific signal increase when using 10% PEG1000, which was used in the 2.3-mPas samples. However, with a higher PEG1000 concentration of 20%, an unspecific signal increase was noticed. With a larger sample size and different analyte concentrations in the main experiment, this effect could potentially be detected at lower PEG1000 concentrations. However, the experiment also showed that the structure can be used to screen LFT buffers because the incubation time remains constant, and thus, the pure effect of the buffer on assay performance can be studied. In summary, the centrifugal cassette offers a solution for enhancing the quantitative resolution of LFTs by offering accurate and fine-tuned incubation times that are independent of the sample viscosity. Table 1 summarizes the most important parameters affecting the centrifugal system and the elimination of the viscosity bias in LFTs.

**Table 1:** Summary of the most important parameters affecting the elimination of the viscosity bias in LFTs

	Parameter	Increase	Decrease
Flow control	Rotational frequency	Flow rate *Q*_*s*_/*Q*_*M,*max_/*Q*_*a*_ increases	Flow rate *Q*_*s*_/*Q*_*M,*max_/*Q*_*a*_ decreases
	Fluidic resistance of transfer channel	Viscosity of sample can influence sample flow rate (liquid viscosity dominated regime)	Lower fluidic resistance guarantees the gas viscosity dominated regime
	Fluidic resistance of venting resistance channel	Flow rate *Q*_*s*_/*Q*_*a*_ decreases (can be compensated by higher rotational frequencies) for medium to high viscosity samples	Flow rate *Q*_*s*_/*Q*_*a*_ increases for low to medium viscosity samples;influence of liquid viscosity increases
	Cross-section (*A*_*m*_)	*Q*_*M,*max_ increases	*Q*_*M,*max_ decreases
	Length of membrane	Dewetting at the radial inward position of the membrane can occur if the centrifugal pressure on the liquid column of the membrane is higher than its capillary pressure	Higher rotational frequencies can be utilized because dewetting at the radial inward position of the membrane is less critical
	Pneumatic air volume	More sample liquid is needed to build up initial pneumatic pressure, risk of bypass due to high flow rates in the initial phase	Less sample liquid is needed to build up initial pneumatic pressure; minimum air volume is defined by the size of the LFS which must be exposed in the chamber to avoid bypass
Immunoassay	Flow rate	Decrease in incubation time results in lower signal intensities because biomolecules have less time to bind to the test line	Longer incubation times cause higher signal intensities because biomolecules have more time to bind to the test line
	Sample volumes	Lower limit of detection due to increased analyte molecules in sample	Higher limit of detection due to decreased analyte molecules in sample

Four potential flow regimes were identified for capillary flow in a tube: gas inertia-dominated, liquid inertia-dominated, gas viscosity-dominated, and liquid viscosity-dominated^22^. To identify the potential flow regimes in the centrifugal cassette, a system-level network simulation^32^ was performed and can be found in detail in the ESI. The simulated pressure evolution of the centrifugal cassette is plotted in ESI Fig. S7. The gas inertia dominates the flow only at the very beginning for approximately 350 ns and can therefore be neglected. The liquid inertia-dominated regime could not be identified because the air inertia of the long venting resistance channel outweighs the initial liquid inertia. Immediately afterward, the gas viscosity-dominated regime takes over. A liquid viscosity-dominated regime could only be observed if the simulation parameters were adjusted. For example, by increasing the acceleration rate to 50 Hz/s and increasing the resistance of the transfer channel by reducing the cross section to 150 × 150 µm, the liquid viscosity dominates for approximately 0.1 s during the buildup of the pneumatic pressure (see ESI Figure S5). In conclusion, only the gas viscosity-dominated regime is of practical relevance for the presented centrifugal cassette.

### Influence of density on flow rate

The driving force of pneumatic flow control in the cassette is the centrifugal force. As shown in Eq. (2), the centrifugal pressure depends on the sample density *ρ*_s_. Hence, the cassette can eliminate viscosity variations, but the flow rate is still susceptible to density variations. The measured densities of the buffers varied slightly: 1.021 g/ml (1.1 mPas), 1.037 g/ml (2.3 mPas), and 1.092 g/ml (24 mPas). According to Eq. (3), the density difference of 7.0% between the low-viscosity buffer and the high-viscosity buffer should result in an increased flow rate. Although there was no significant difference between the flow rates of the 1.1-mPas sample and the 24-mPas sample (*p* = 0.259), the measured increase in the mean flow rate of 4.4%, from 2.98 µl/min for the 1.1-mPas sample to 3.11 µl/min for the 24-mPas sample could indicate a higher flow rate due to an increased density.

However, body fluids are usually aqueous, and their densities are comparable to that of water. For example, the density of unstimulated total saliva is 1.002–1.012 g/ml^33^, that of blood plasma is 1.025–1.034 g/ml^34^, and that of common urine is 1.010–1.020 g/ml^35,36^. Compared with possible variations in the viscosity of biological samples, the variations in density are negligible.

### Influence of ambient temperature

When using pneumatic flow control, the flow rate through the membrane is in theory less affected by any ambient temperature change because temperature affects the viscosity of air much less than it affects the viscosity of water-based solutions. For example, if the temperature is increased from 15 °C to 35 °C, the viscosity of water decreases by 37% (from 1.13 mPas to 0.71 mPas)^37^. In contrast, the viscosity of air at 50% relative humidity increases by just 3% (from 0.0178 mPas to 0.0183 mPas)^38^. The reduced temperature dependence is an additional feature of the structure but is not explicitly covered in this study. Nevertheless, this could be an additional advantage for field applications.

### Limitations of the current structure

The current structure was designed to work with typical lateral flow assay components, and its functionality is shown above. Nevertheless, we identified two challenges with the current design that may occur if processing parameters are changed or if different liquids are used. The first challenge is in the case of an intended discontinuous application of liquid onto the membrane. If the rotation stops, the liquid flow into the pneumatic chamber also stops, and the overpressure is relieved. If the rotation and the liquid flow are then started again, the flow rate is initially higher since the overpressure in the pneumatic chamber must be built up first. This increased liquid inflow at the very beginning of the test was easily absorbed by the dry membrane. However, if the membrane is already filled, the maximum flow rate through the membrane at a certain rotational frequency is restricted due to viscous dissipation in the membrane. If the flow rate onto the membrane is higher than the maximum possible flow rate through the membrane, bypass occurs, as shown earlier^26^.

The second challenge is the transition between the transfer channel and the pneumatic chamber. Only a small amount of centrifugal pressure was needed to achieve relevant flow rates using the cassette. In our experiments, 13.7 hPa was enough pressure to attain a flow rate of approximately 3.0 µl/min. A discontinuous, dripping flow of liquids onto the membrane may result in bypass if the resulting droplet volume is too high. Using pure water, for example, can lead to a discontinuous flow onto the membrane. Generally, there are detergents in an LFT running buffer to prevent unspecific binding. However, detergents also reduce the surface tension of the sample and lead to continuous flow onto the membrane. Therefore, 1% Tween 20 was added to all buffers used in the presented experiments. If no detergents can be used in the assay, the design of the cross-section widening from the transfer channel into the pneumatic chamber must be optimized.

During liquid transfer, the radial fill height of the inlet chamber decreases, which results in a decrease in Δ*p*_cent_. In the current design, the centrifugal pressure decreases from 13.7 hPa (chamber filled) to 6.1 hPa (chamber completely empty). Since centrifugal pressure is the driving force of the system, the flow rate also decreases over time. However, this effect has no influence on the general functionality of the structure because only the maximum flow rate at the beginning must be known in order to prevent bypass. If a constant flow rate over time is desired, the chamber design could be optimized by using a deeper, wider chamber in an isoradial direction. The decreasing hydrostatic height could be compensated by increasing the frequency over time to maintain a constant centrifugal pressure.

## Conclusion

LFTs are fantastic point-of-care tests. However, varying sample viscosities are a challenge for quantitative LFTs because the flow rate through the strip depends on the sample viscosity. Inconsistent flow rates lead to a signal-intensity bias due to varying incubation times and thus alter the quantitative results. This phenomenon limits the applications of LFTs by narrowing the set of usable sample matrices.

In this work, sample viscosity-independent flow through an LFT was demonstrated by using a centrifugal microfluidic-driven cassette for the LFT. The centrifugal cassette almost completely eliminates the viscosity bias of lateral flow assays. The elimination of viscosity bias opens up new fields of application that require the development of highly sensitive and quantitative assays with sample matrices that have a wide range of viscosities.

## Materials and methods

### Fabrication and design of the centrifugal cassette

A centrifugal cassette with pneumatic flow control was developed to address the problem of viscosity variation and is shown in Fig. 2b. The venting resistance channel was 60-µm wide and 47-µm high. The maximum length of the channel was 416 mm, but this length could be reduced by opening optional vents along the channel. For the experiments in this study, the maximum length of the venting resistance channel was used. The inlet chamber was always filled with a sample volume of 30 µl. The resulting radial inner position of the liquid column *r*_1_ was 43.5 mm, and the radial outer position *r*_2_ was 46.9 mm.

The centrifugal cassettes were fabricated as CD-sized foil disks by the Hahn–Schickard Lab-on-a-Chip Foundry using microthermoforming^39^. The computer-aided design was performed using SolidWorks (Dassault Systèmes, France). The foil disk material used was a 188-µm-thick cyclo olefin polymer foil ZF 14 (Zeon Chemicals, USA). The membrane strips were glued into structured disks at a radial position *r*_in,M_ of 49.5 mm with double-sided adhesive tape (3 M 8153 300 LSE, USA). Afterward, the disks were sealed with pressure-sensitive adhesive foil 9795 R (3 M, USA). Then, the sealed disks were cut to their final shape with a CO_2_ laser.

### Lateral flow assay and processing

For the human IgG lateral flow assay, nitrocellulose CN140 (Sartorius, Germany) was used as the chromatographic membrane. For experiments using the centrifugal cassette, the membrane was glued onto a double-sided adhesive sheet (3 M 8153 300 LSE, USA). Dipstick LFTs were used for the reference experiments. For these, a 20-mm CN140 membrane strip was glued onto a PVC backing card (Kenosha, Netherlands) with a CF5 absorption pad (GE Healthcare Life Sciences, USA).

A human IgG line was printed onto all CN140 membranes using a BioSpot BT600 (Biofludix, Germany). The printing solution was a water-based solution with 0.1 mg/ml IgG from human serum (Sigma–Aldrich, USA), 0.1% heat-shocked BSA (Sigma–Aldrich, USA), and 0.05% trehalose (Sigma–Aldrich, USA). The displacement between the printed 30-nl droplets was adjusted to achieve a printing volume of 1 µl/cm.

For the experiments in the centrifugal cassette, the membrane sheets were stamped to a final size of 4.4 mm by 8 mm. The dipsticks were cut with a guillotine cutter (Arista Biologicals, USA) to a width of 4.4 mm.

The analyte for the model assay was human IgG (Sigma–Aldrich, USA). Therefore, a dilution row with the following concentrations was prepared: 0, 1, 10, 10^2^, 10^3^, 10^4^, and 10^5^ pg/ml. The sample buffer was PBS (Thermo Fisher Scientific Inc., USA) with 3% heat-shocked BSA (Sigma–Aldrich, USA), 1% Tween 20 (Carl Roth, Germany), and goat anti-human IgG 40-nm gold nanoparticles (BBI Solutions, United Kingdom) with an optical density of 0.4. To increase the viscosity of the sample buffer, 10% PEG1000 (Merck KGaA, Germany) was added to achieve a viscosity of 2.3 mPas. Samples with a viscosity of 24 mPas were not used for the immunoassay because only samples with a viscosity less than 2.35 mPas can be transferred through the membrane bypass free using the presented centrifugal cassette (see calculation of *Q*_*M*,max_ in Eq. (1)). Therefore, the 24-mPas PBS-based buffer consisted only of 50% PEG1000 and 1% Tween 20. The viscosities were measured at 24 °C using an MCR101 rheometer (Anton Paar GmbH, Germany). The densities of the samples were determined gravimetrically.

Thirty microliters of each sample was manually pipetted into a structure on the centrifugal cassettes. The cassettes were processed with a centrifugal setup custom-built by QIAGEN Lake Constance, Germany. The rotational frequency was increased from 0 Hz to 15 Hz at 0.5 Hz/s and then held constant. After 10 min, the membranes were drained by increasing the frequency to 75 Hz at 1 Hz/s. The total flow time through the structure was measured as follows: the starting point was when the constant processing frequency (15 Hz) was reached, and the endpoint was when the inlet chamber was emptied during the first 10 min. The mean flow rate was calculated by dividing the volume of the sample still in the inlet chamber at the starting point by the total flow time. The human IgG assay was performed three times for each concentration, resulting in 21 flow-rate measurements for each viscosity. The total flow time of the 24-mPas sample was measured eight times.

The dipstick assay was performed in a microtiter plate: 30 µl of sample was pipetted into a well on a 96-microtiter plate, and then the dipstick was inserted. After 10 min, the dipsticks were removed from the microtiter plate. The waste pad was removed to minimize drying effects. Three dipstick assays were performed for each concentration.

All experiments were performed at room temperature. Statistical evaluation of the flow-rate data was performed in MiniTab using ANOVA and Student’s *t*-test.

### Analysis of the strip

The membranes of the centrifugal cassette and the dipsticks without waste pads were dried overnight at room temperature in a drying box with silica beads. The dried membranes were then scanned at 1200 dpi using a v39 precision office scanner (Epson, Japan). The test line was then analyzed using an automated Python peak analyzing script. The PeakUtils library was used to perform the baseline correction and to find peaks. The height of the peak represents the peak intensity. For comparison, the peak intensity of the test line was set in relation to the peak intensity of the 0 ng/ml sample with a viscosity of 1.1 mPas. Therefore, the final signal intensity is given as a percent.

## Supplementary information

Eliminating viscosity bias in lateral flow strips - ESI
