# Supplementary material for: Eliminating viscosity bias in lateral flow tests
Source: Microsyst Nanoeng. 2021 Sep 6;7:72. doi: 10.1038/s41378-021-00296-5 (PMC8433459; doi:10.1038/s41378-021-00296-5)
Supplement: Supplementary file 1 — Eliminating viscosity bias in lateral flow strips - ESI [file 41378_2021_296_MOESM1_ESM.docx]

Electronic supplementary information

# Eliminating viscosity bias in lateral flow strips

Daniel M. Kainz,^1,2^ Bastian J. Breiner,^2^ Susanna M. Früh,^1,2^ Tobias Hutzenlaub,^1,2^ Roland Zengerle,^1,2^ and Nils Paust^1,2^

1: Laboratory for MEMS Applications, IMTEK - Department of Microsystems Engineering, University of Freiburg, Georges-Koehler-Allee 103, 79110 Freiburg, Germany

2: Hahn-Schickard, Georges-Koehler-Allee 103, 79110 Freiburg, Germany

### **Design guideline for lateral flow strip integration**

According to our previously published design guideline, the maximum flow rate *Q_M,max_* through the exposed membrane in a centrifugal cassette without bypass can be determined with the following equation:^1^

| $Q_{M,max}=\frac{\rho_{s}*r_{in,M}*\omega^{2}*A_{M}*\kappa}{\eta_{s}}$ | (1) |
| --- | --- |

Where *ρ_s_* denotes the density of the sample, *r_in,M_* the inner radial position of the membrane, *ω* the angular frequency, *A_M_* the cross-sectional area of the membrane, *κ* the permeability of the membrane and *η_s_* the viscosity of the sample.

To prevent bypass, the sample inflow *Q_s_* into the pneumatic chamber must be lower than or equal to the maximum possible flow rate *Q_M,max_* through the membrane:

| $Q_{s}\leq Q_{M,max}$ | (2) |
| --- | --- |

To calculate *Q_s_* we developed a simplified model of the structure. We assume that in the ideal state of equilibrium, the pneumatic pressure *Δp_pneu_* in the pneumatic chamber is equal to the centrifugal pressure *Δp_cent_* in the liquid column of the inlet chamber and transfer channel and equal to the pressure drop *Δp_visc,R_* in the venting resistance channel due to viscous dissipation:

| $\vert{\Delta p}_{cent}\vert=\vert{\Delta p}_{pneu}\vert=\vert{\Delta p}_{visc,R}\vert$ | (3) |
| --- | --- |

The viscous dissipation in the transfer channel is deemed negligible at low flow rates due to the low fluidic resistance of the transfer channel. The compression of air inside a channel and the time-dependent change of liquid volumes in chambers are also not considered.

The centrifugal pressure *Δp_cent_* is the driving force in the system and can be calculated as follow:

| ${\Delta p}_{cent}=\frac{\rho_{s}}{2}\omega^{2}\left( {r_{2}}^{2}-{r_{1}}^{2} \right)$ | (4) |
| --- | --- |

Where r_2_ denotes the outer radial position of the channel and r_1_ the inner radial position of the liquid column in the inlet chamber.

The viscous dissipation *Δp_visc,R_* in the venting resistance channel can be calculated as:

| ${\Delta p}_{visc,R}=Q_{R}C_{g}\frac{\eta_{a}l_{R}}{{A_{R}}^{2}}$ | (5) |
| --- | --- |

Where *Q_R_* denotes the air flow rate through the venting resistance channel, *C_g_* the geometry factor of the channel, *η_a_* the viscosity of air, *l_R_* the length of the venting resistance channel and *A_R_* the cross-sectional area of the air resistance channel. Different channel profiles have different geometry factors which can be found in the references.^2–4^

Being in the equilibrium state, eqn (4) and eqn (5) can be inserted into eqn (3) and solved for the flow rate *Q_R_*:

| $Q_{R}=\frac{\frac{\rho_{s}}{2}\omega^{2}\left( {r_{2}}^{2}-{r_{1}}^{2} \right)}{C_{g}\frac{\eta_{a}l_{R}}{{A_{R}}^{2}}}$ | (6) |
| --- | --- |

To have a constant pneumatic pressure in the pneumatic chamber in the state of equilibrium, the sample inflow *Q_s_* must be equal to the air outflow *Q_R_* through the resistance channel:

| $Q_{s}=Q_{R}=\frac{\frac{\rho_{s}}{2}\omega^{2}\left( {r_{2}}^{2}-{r_{1}}^{2} \right)}{C_{g}\frac{\eta_{a}l_{R}}{{A_{R}}^{2}}}$ | (7) |
| --- | --- |

where *Q_s_* denotes the sample flow rate through the transfer channel. According to eqn.(7), *Q_s_* depends only on the viscosity of air and is independent of the sample viscosity.

Similar to our previous study, a design factor *D* can be introduced to describe the microfluidic structure.^1^ *D* is defined as the ratio of *Q_s_* and *Q_M,max_* and should be less than or equal to 1 to prevent bypass:

| $D=\frac{Q_{s}}{Q_{M,max}}\leq1$ | (8) |
| --- | --- |

Inserting eqn (7) and eqn (1) into eqn (8), the final equation for the design factor *D* to prevent bypass can be written as:

| $D=\frac{\eta_{s}({r_{2}}^{2}-{r_{1}}^{2})}{{2*C}_{g}\frac{\eta_{a}l_{R}}{{A_{R}}^{2}}*r_{in,M}*A_{M}*\kappa}\leq1$ | (9) |
| --- | --- |

Despite a sample viscosity independent inflow into the pneumatic chamber, the membrane restricts the maximal possible sample viscosity to assure a bypass free flow through the membrane.

### **Pre-experiments for determining suitable PEG1000 concentration**

The influence of PEG 1000 on the human IgG assay performance was tested. Different concentrations of PEG 1000 were added to into the buffer and the sample was processed in the centrifugal cassette. The sample was a negative control with no added analyte (see Table S1).

Table S1: Scanned LFTs with different PEG 1000 concentrations in the buffer. All strips were processed in the centrifugal cassette to minimize viscosity bias. The line intensity is the measured peak height of the profile plot (ImageJ) of the line. The background was substracted.

| **Reference sample** | **10 % PEG 1000 in sample** | **20 % PEG 1000 in sample** |
| --- | --- | --- |
| 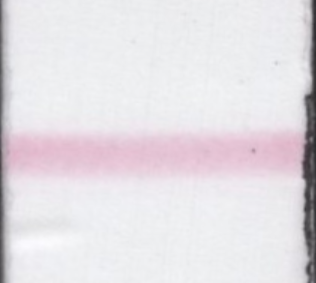 | 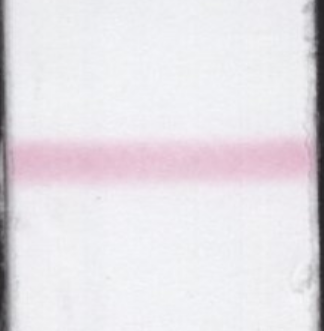 | 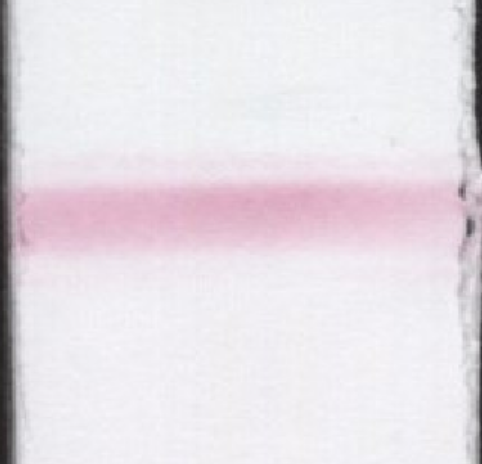 |
| Line intensity: 28.8 a.u. | Line intensity: 29.0 a.u. | Line intensity: 40.1 a.u. |
|  |  | *Unspecific binding to the test line due to high PEG 1000 concentration* |

### **System-level network simulation**

The previously published system-level network simulation^5^ was used to identify the flow regimes. First, a radial channel connected to an inlet chamber was simulated at a constant frequency. Afterwards, the influence of an initial frequency-accelerating phase on the radial channel system was analysed. Finally, the presented centrifugal cassette was simulated and the influence of rotational acceleration was analysed as well as the fluidic resistance of the transfer channel and the pneumatic chamber volume. The utilization of the network simulation is explained in detail by Hess et al. and Schwarz et al.^5,6^ Euler force, Coriolis force, compression of air in channels and capillary forces are neglected. Chambers are simulated without inertia forces. In addition, the liquid fill level of the inlet chamber is not changing over time to have a constant centrifugal pressure by the chamber.

A fluidic network model was built for the inlet chamber which consists of a chamber and a radial channel as displayed in Figure S1. The model was simulated with a constant frequency of 15 Hz and afterwards with an acceleration phase from 0 Hz to 15 Hz with 15 Hz/s. The simulated results are plotted in Figure S2 and Figure S3.


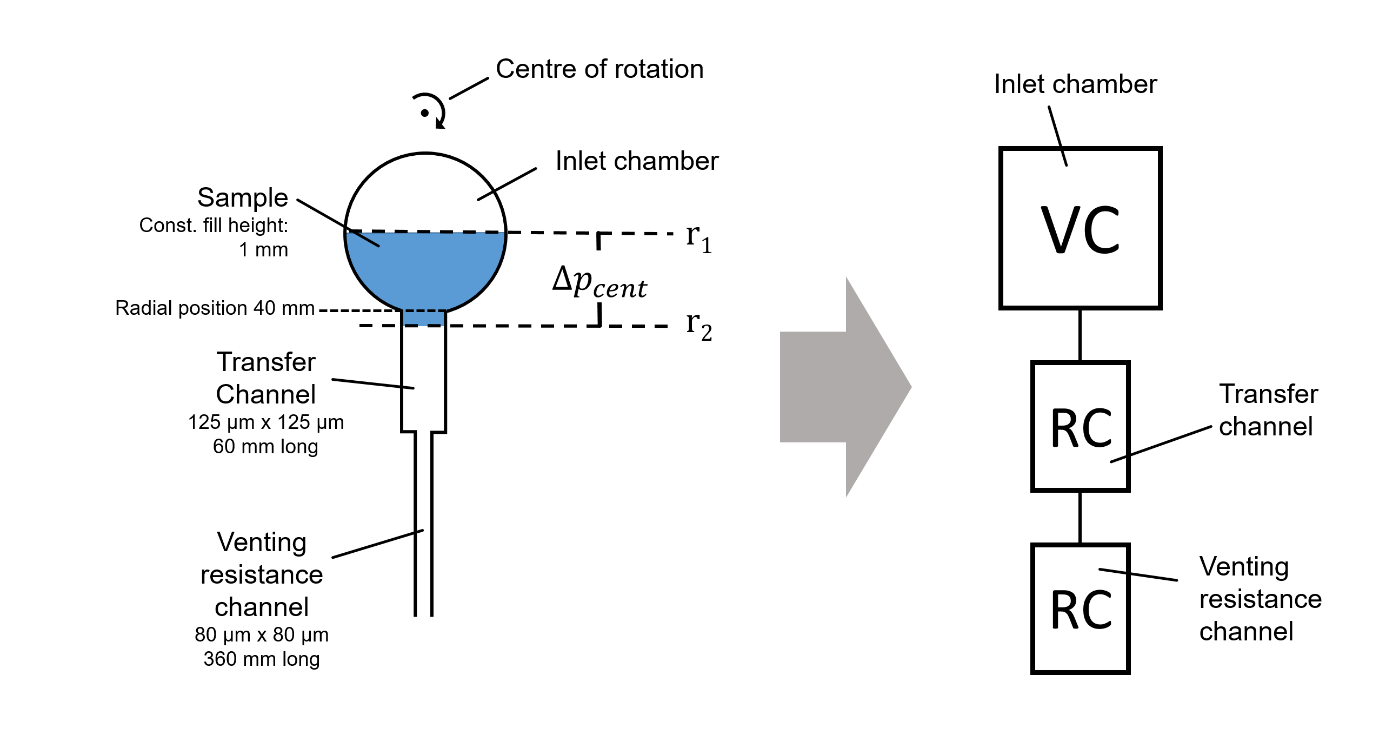


Figure S1: Schematic illustration of the centrifugal structure (left) and fluidic network model (right). Used elements are vented chamber (VC) and radial channels (RC). The elements are connected (lines) in the fluidic network model as indicated.

According to the simulation, the flow starts in the gas inertia dominated regime. Due to the long channel filled with air, the gas inertia dominated regime outweighs the liquid dominated regime completely. In contrast to the capillary driven flow in a tube, no liquid inertia dominated regime could be observed before the gas viscosity dominated regime was entered. Gradually, the radial channel is filled due to liquid flow. Thus, the centrifugal pressure (driving force of the system) is increasing with the increasing liquid column height (*r_2_­r_1_*). The flow rate further increases and the system is accelerating itself. By increasing the liquid column height, the liquid viscous dissipation increases until it outweighs the gas viscous dissipation, entering the final liquid viscosity dominated regime at around 0.88 s.


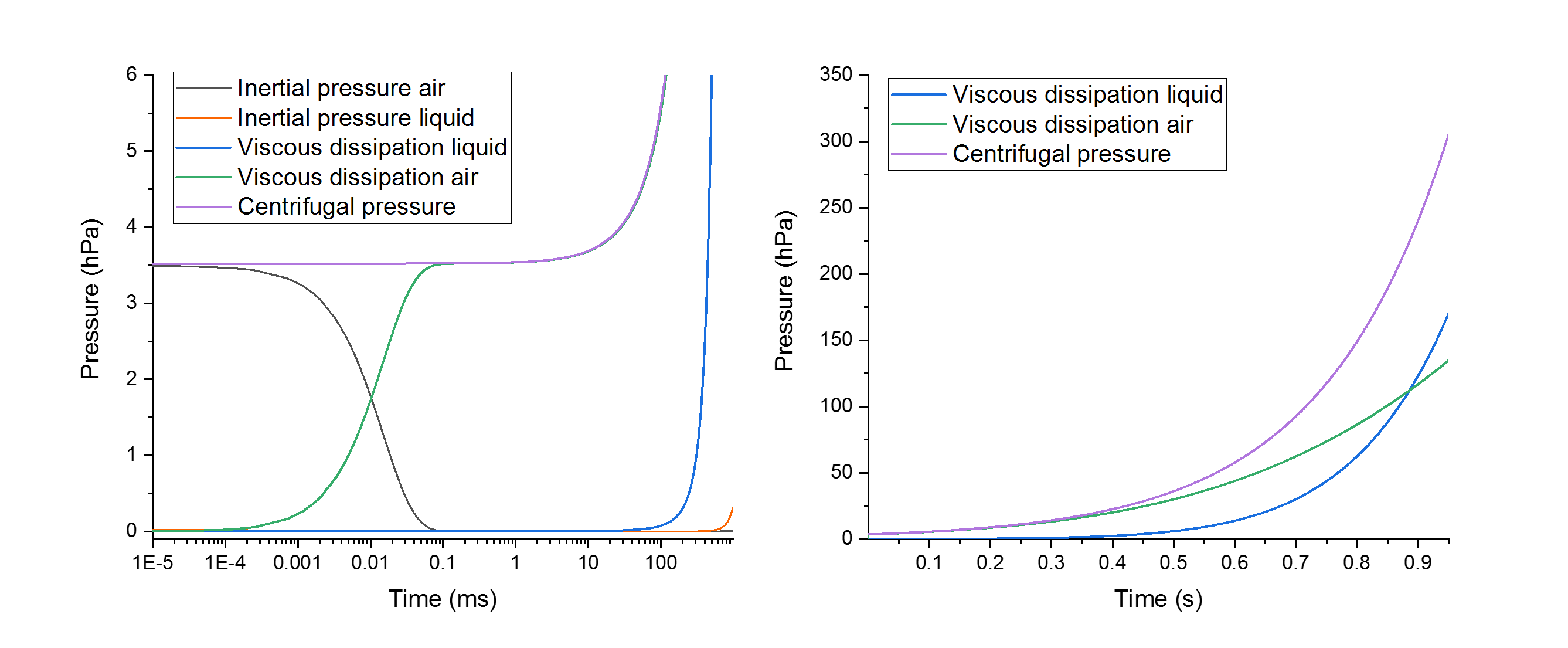


Figure S2: Simulation results of the chamber with connected radial channel at a constant frequency. At the beginning, the gas inertia dominated regime can be observed (left). Afterwards, the flow is dominated by the air viscosity dominated regime, until the liquid viscosity dominated regime takes over at around 0.88 s (right).

In the next simulation, the centrifugal pressure starts at 0 Hz and is accelerated with 15 Hz/s to 15 Hz. The simulation results can be seen in Figure S3. With increasing frequency, the centrifugal pressure increases and the fluids start to flow. During the acceleration phase, the frequency increase as well as the increase of the liquid column contribute to an acceleration of the centrifugal pressure. At time point 1 s, 15 Hz are reached and a change in the slope of the centrifugal pressure can be seen because of the missing increase in frequency. In contrast to a constant frequency from the beginning, the air inertial pressure loss ramps up with increasing centrifugal pressure, but is quickly outweighed by the gas viscous dissipation. Similar to the previous simulation, the liquid viscosity regime dominates at the end.


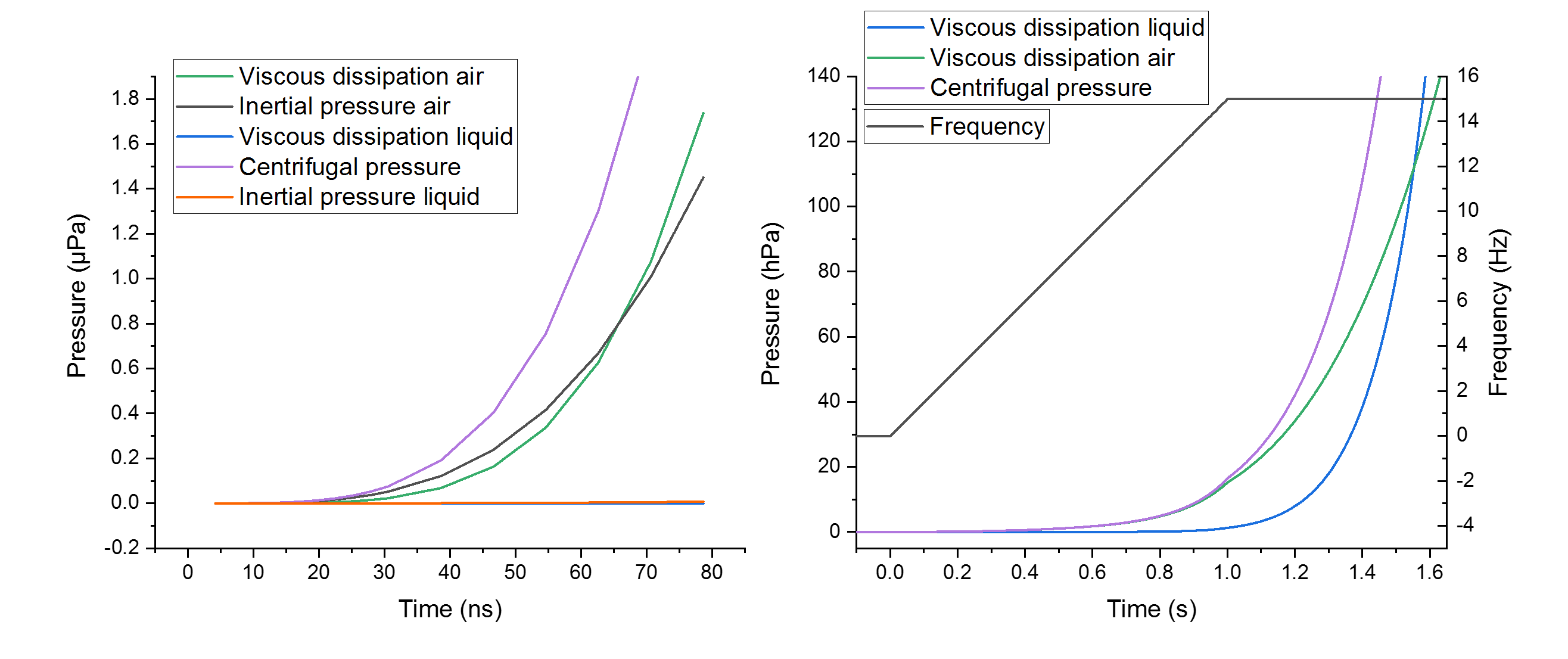


Figure S3: Results of the simulation of the chamber with connected radial channel. The frequency is accelerated to 15 Hz within 1 s. At the beginning, the gas inertia dominated regime can be observed but is quickly outweighed by the air viscosity regime (left). After the air viscosity dominated regime, the liquid viscosity dominated regime dominates at the end (right).

The network simulation model of the centrifugal cassette is illustrated in Figure S4. The design parameters of the cassette were modified in this example. The transfer channel cross section was decreased to 150 µm x 150 µm and the pneumatic chamber volume was increased to 100 µm. The frequency is accelerated in case A) with 50 Hz/s and in case B) with 2.5 Hz/s to 5 Hz to analyse the dynamic character of the system. Both simulation results can be seen in Figure S5. The simulation results of the original described centrifugal cassette design can be found in Figure S7.


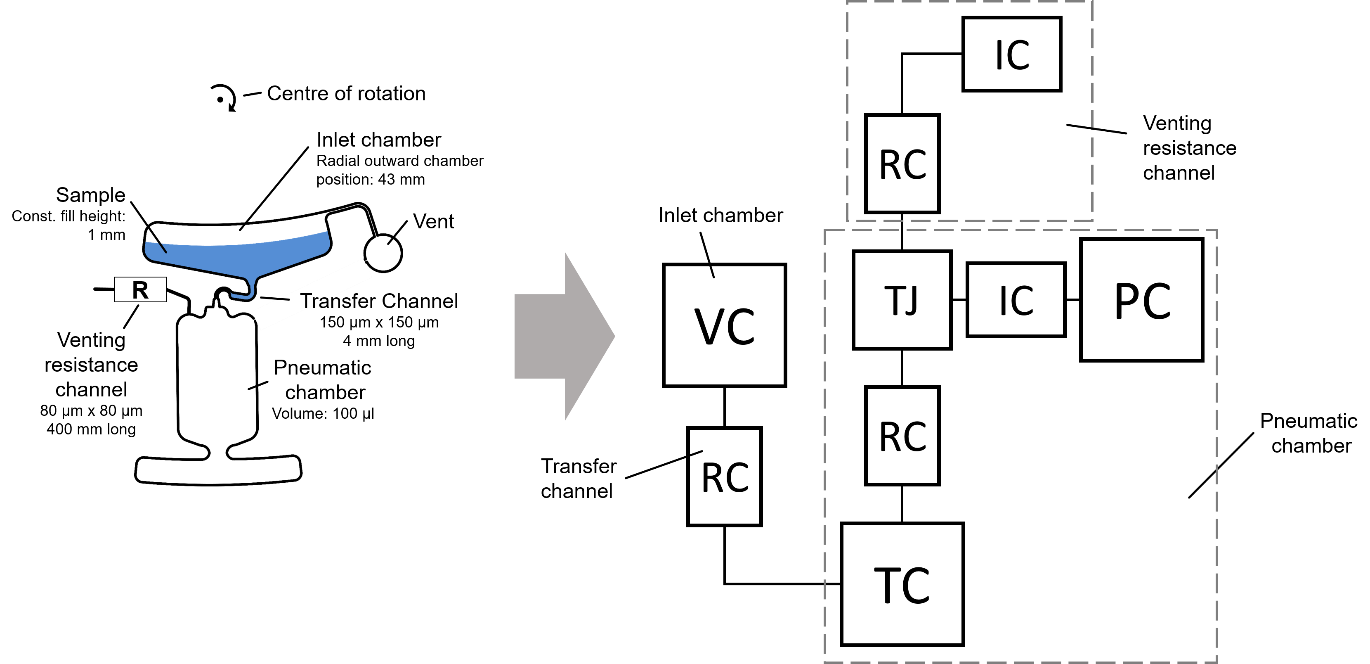


Figure S4: Structure of the centrifugal cassette (left) and fluidic network model (right). The elements are vented chambers (VC), radial (RC) and isoradial channels (IC), two-port chambers (TC), T‑junctions (TJ) and pneumatic chambers (PC). The elements are connected (lines) in the fluidic network model as indicated.

Within the first 0.2 s, case A) seems to behave like the simulation of the radial channel: the gas inertia regime dominates at first, followed by the gas viscosity dominated regime and afterwards by a liquid viscosity dominated regime. After reaching the target frequency, the centrifugal pressure stays constant as soon as the transfer channel is completely filled, in contrast to the simulation of the radial channel. Liquid which is transferred further, is collected in the pneumatic chamber and does not increase the liquid column height. The transferred liquid builds up the pressure in the pneumatic chamber. With increasing pneumatic pressure, the air flow rate through the resistance channel increases and thus the viscous dissipation of air increases. Since the centrifugal pressure is limited, the liquid viscous dissipation decreases and thus the liquid flow rate in the transfer channel decreases and the gas viscosity dominated regime is entered. In the equilibrium phase, the pneumatic pressure and the pressure loss due to the viscous dissipation of air are equal but less than the centrifugal pressure. The remaining centrifugal pressure is lost due to the viscous dissipation of liquid in the transfer channel. This result stresses the need of a transfer channel with a low fluidic resistance to minimize sample viscosity effects.


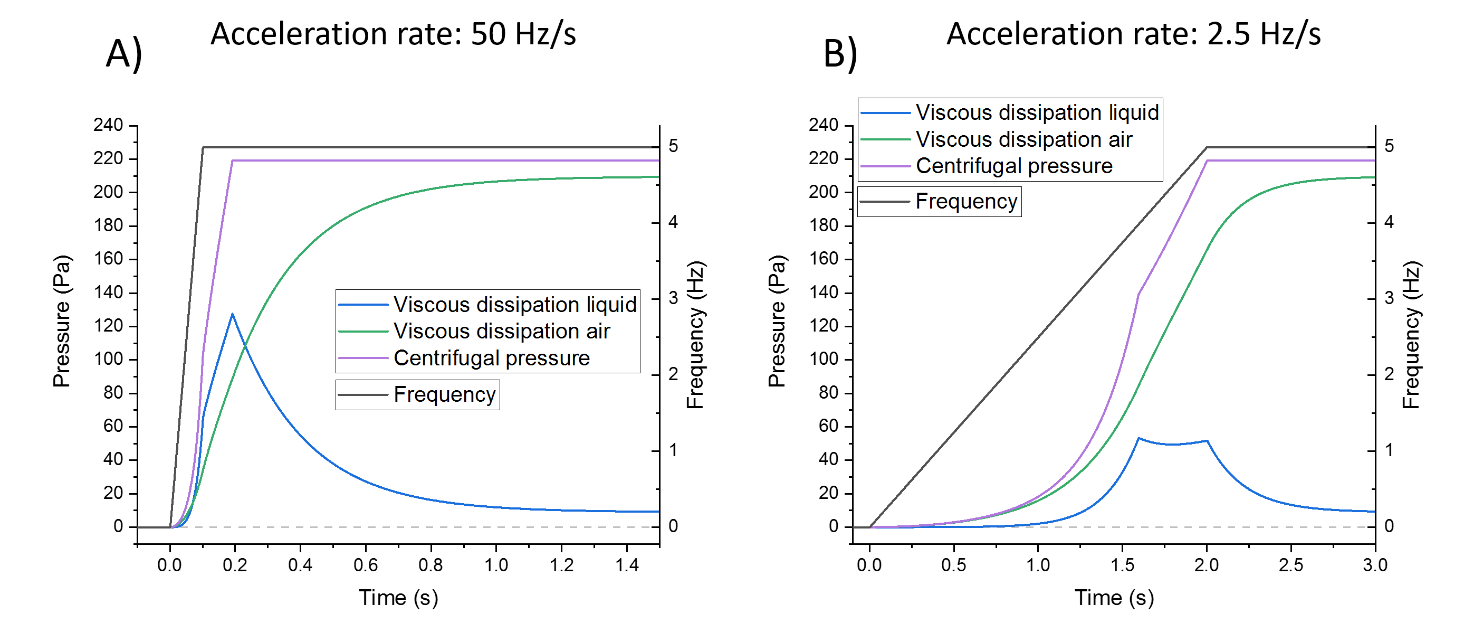


Figure S5: Results of the simulation of the centrifugal cassette. The cassette was accelerated with 50 Hz/s (A) and with 2.5 Hz/s (B). The acceleration rate influences the intensity of the liquid viscous dissipation pressure loss during the build up of the pneumatic pressure. At high acceleration rates, an intermediate liquid viscosity dominated regime is formed.

In case B) (low acceleration rate of 2.5 Hz/s), the transfer channel is filled with liquid before the maximum frequency is reached and explains the change in slope of the centrifugal pressure curve at around 1.5 s. In contrast to the high acceleration rate, the flow rate and thus the liquid viscous dissipation in the transfer channel never overcomes the viscous dissipation of air. The liquid viscous dissipation can be even further decreased by decreasing the transfer channel fluidic resistance and by decreasing the pneumatic chamber volume as shown in Figure S6.

The simulation results of the original design of the centrifugal cassette are plotted in Figure S7. Therefore, the rotational acceleration rate was even further decreased to 1 Hz/s. The target frequency was 15 Hz. For approximately 350 ns the gas inertia dominated regime is the prominent regime. Afterwards, the complete flow is controlled by the gas viscosity dominated regime. According to the simulation, no liquid dominated regime can be observed using the presented centrifugal cassette.


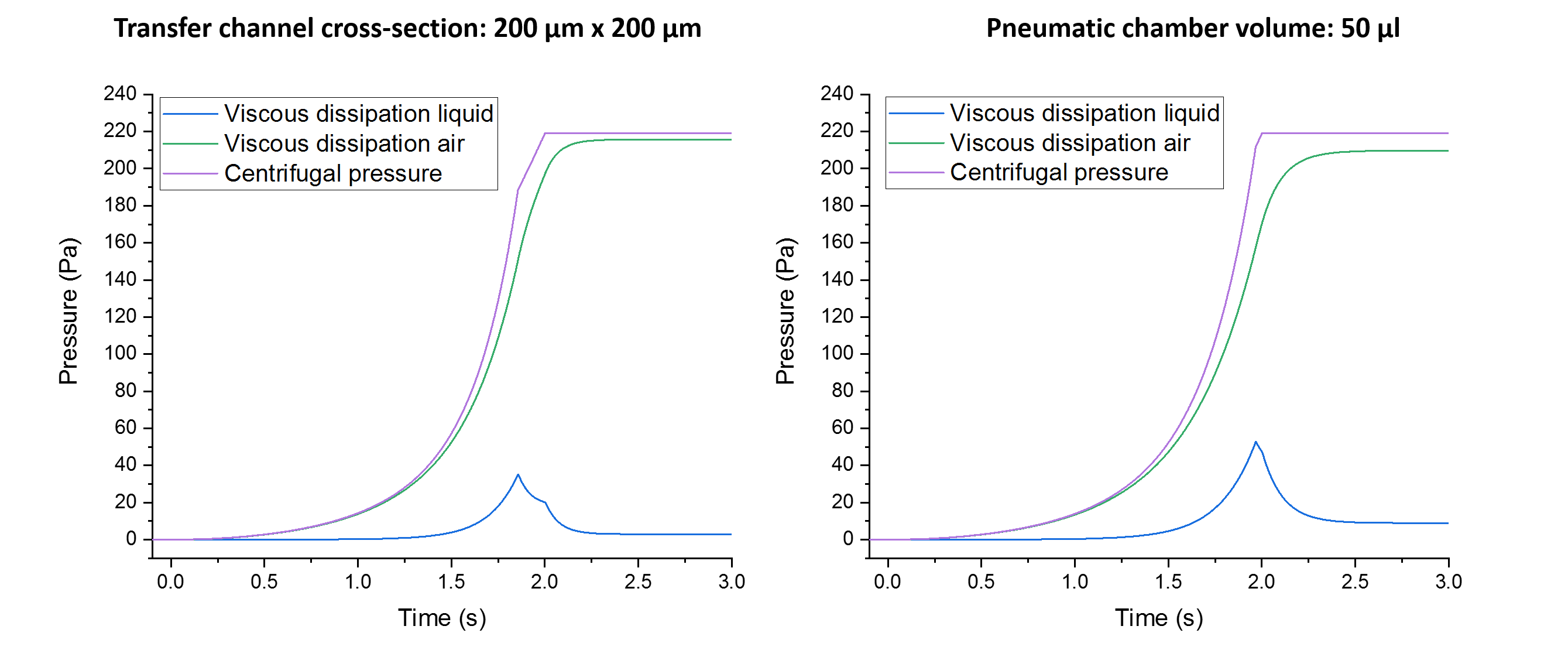


Figure S6: Reduction of liquid viscous dissipation by increasing the transfer channel cross-section from 150 µm x 150 µm to 200 µm x 200 µm to reduce the fluidic resistance (left) and by decreasing the pneumatic chamber volume from 100 µl to 50 µl.


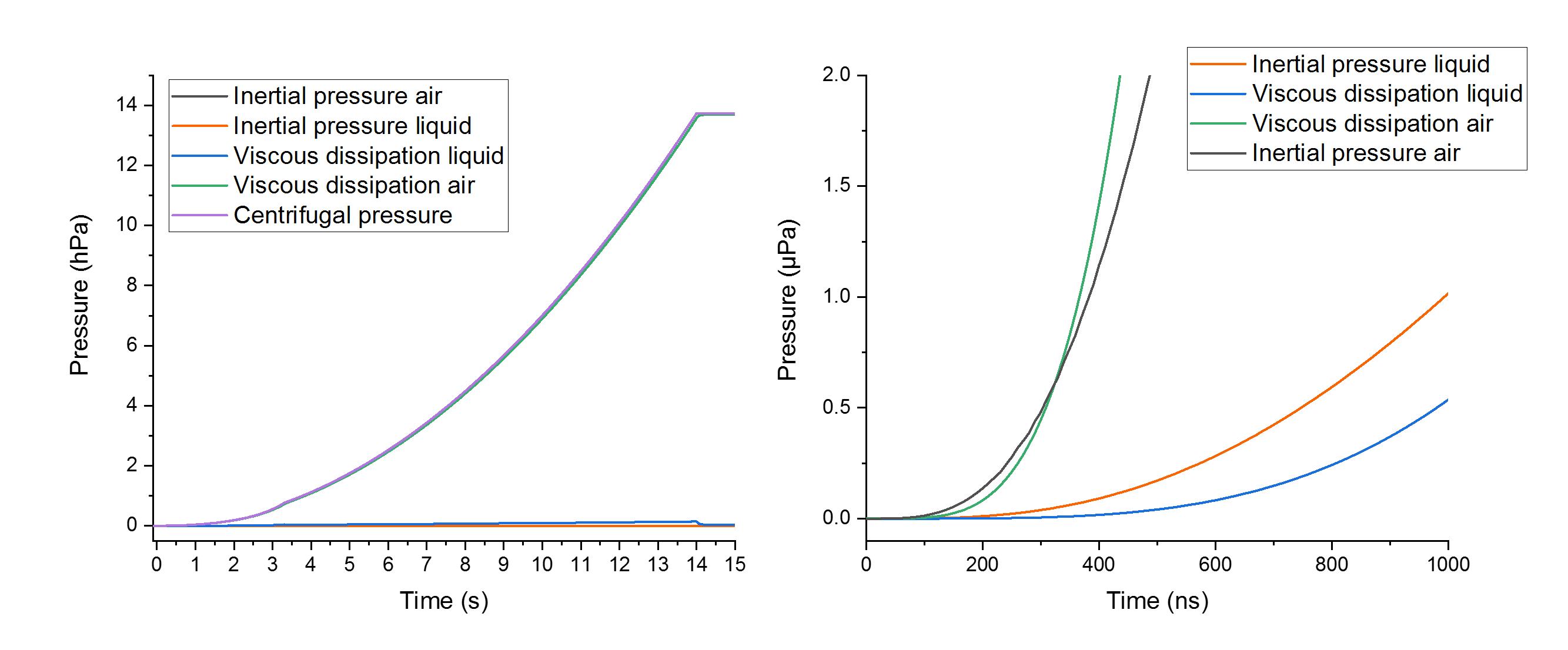


Figure S7: Simulation results of the original centrifugal cassette design with the sample viscosity 2.3 mPas and an acceleration phase to 15 Hz with 1 Hz/s. Nearly no liquid viscous dissipation can be observed during the acceleration phase and in the final equilibrium state (left). The first dominating regime is the gas inertia regime, directly followed by the gas viscosity dominated regime (right).

References

1. Kainz, D. M., Früh, S. M., Hutzenlaub, T., Zengerle, R. & Paust, N. Flow control for lateral flow strips with centrifugal microfluidics. *Lab on a chip* **19,** 2718–2727; 10.1039/C9LC00308H (2019).

2. Bahrami, M., Yovanovich, M. M. & Culham, J. R. Pressure Drop of Fully-Developed, Laminar Flow in Microchannels of Arbitrary Cross-Section. *J. Fluids Eng.* **128,** 1036; 10.1115/1.2234786 (2006).

3. Bruus, H. *Theoretical microfluidics* (Oxford Univ. Press, Oxford, 2008).

4. Richter, M., Woias, P. & Weiβ, D. Microchannels for applications in liquid dosing and flow-rate measurement. *Sensors and Actuators A: Physical* **62,** 480–483; 10.1016/S0924-4247(97)01486-6 (1997).

5. Schwarz, I., Zehnle, S., Hutzenlaub, T., Zengerle, R. & Paust, N. System-level network simulation for robust centrifugal-microfluidic lab-on-a-chip systems. *Lab on a chip* **16,** 1873–1885; 10.1039/C5LC01525A (2016).

6. Hess, J. F. *et al.* Review on pneumatic operations in centrifugal microfluidics. *Lab on a chip* **19,** 3745–3770; 10.1039/C9LC00441F (2019).
